# RADIA: RNA and DNA Integrated Analysis for Somatic Mutation Detection

**DOI:** 10.1371/journal.pone.0111516

**Published:** 2014-11-18

**Authors:** Amie J. Radenbaugh, Singer Ma, Adam Ewing, Joshua M. Stuart, Eric A. Collisson, Jingchun Zhu, David Haussler

**Affiliations:** 1 University of California Santa Cruz Genomics Institute, Department of Biomolecular Engineering, University of California Santa Cruz, Santa Cruz, California, United States of America; 2 Division of Hematology/Oncology, University of California San Francisco, San Francisco, California, United States of America; 3 Howard Hughes Medical Institute, Chevy Chase, Maryland, United States of America; H. Lee Moffitt Cancer Center & Research Institute, United States of America

## Abstract

The detection of somatic single nucleotide variants is a crucial component to the characterization of the cancer genome. Mutation calling algorithms thus far have focused on comparing the normal and tumor genomes from the same individual. In recent years, it has become routine for projects like The Cancer Genome Atlas (TCGA) to also sequence the tumor RNA. Here we present RADIA (RNA and DNA Integrated Analysis), a novel computational method combining the patient-matched normal and tumor DNA with the tumor RNA to detect somatic mutations. The inclusion of the RNA increases the power to detect somatic mutations, especially at low DNA allelic frequencies. By integrating an individual’s DNA and RNA, we are able to detect mutations that would otherwise be missed by traditional algorithms that examine only the DNA. We demonstrate high sensitivity (84%) and very high precision (98% and 99%) for RADIA in patient data from endometrial carcinoma and lung adenocarcinoma from TCGA. Mutations with both high DNA and RNA read support have the highest validation rate of over 99%. We also introduce a simulation package that spikes in artificial mutations to patient data, rather than simulating sequencing data from a reference genome. We evaluate sensitivity on the simulation data and demonstrate our ability to rescue back mutations at low DNA allelic frequencies by including the RNA. Finally, we highlight mutations in important cancer genes that were rescued due to the incorporation of the RNA.

## Introduction

Much of our current understanding of cancer has come from investigating how normal cells are transformed into cancerous cells through the stepwise acquisition of somatic genomic abnormalities. These events include point mutations, insertions and deletions (INDELs), chromosomal rearrangements, and changes to the copy number of segments of DNA. Transforming a normal human cell into a malignant, immortal cancer cell line requires an estimated five to seven genetic alterations in key genes and pathways [1], [2]. Not surprisingly, much research has been devoted to determining how cancer cells are able to acquire their abilities through the accumulation of somatic mutations.

The Cancer Genome Atlas (TCGA) project has produced exome-wide data from thousands of tumors and patient-matched normal tissues. With the development of RNA Sequencing (RNA-Seq) [3], TCGA began providing an additional high-throughput tumor sequence dataset. These three datasets consisting of tumor and patient-matched normal DNA and tumor RNA have become a new standard in cancer genomics. RNA-Seq enables one to investigate the consequences of genomic changes in the RNA transcripts they encode to better characterize 1) germline variants, 2) somatic mutations, and 3) variants in the RNA that are not found in the DNA that could be the result of RNA editing [4].

Over the next few years, many more whole-genome and exome-capture DNA and RNA-Seq BAM (the binary version of Sequence Alignment/Map [5]) files will become available. TCGA has collected over 10,000 tissue samples from more than 20 types of cancer. There is a clear need for an efficient method for the combined analysis of patient-matched tumor DNA, normal DNA, and tumor RNA. Here we present a method called RADIA to identify and characterize alterations in cancer using DNA and RNA obtained by high-throughput sequencing data.

Somatic mutation calling is traditionally performed on patient-matched pairs of tumor and normal genomes/exomes [6]–[11]. The ability to accurately detect somatic mutations is hindered by both biological and technical artifacts that make it difficult to obtain both high sensitivity and high specificity. Different mutation calling algorithms often disagree about putative mutations in the same source data, and frequently have discernible systematic differences due to the trade-off between sensitivity and specificity [12]. This is especially true for somatic mutations with low variant allele frequencies (VAFs). By creating an algorithm that utilizes both DNA and RNA, we have increased the power to detect somatic mutations, especially at low variant allele frequencies.

**Figure 1:**
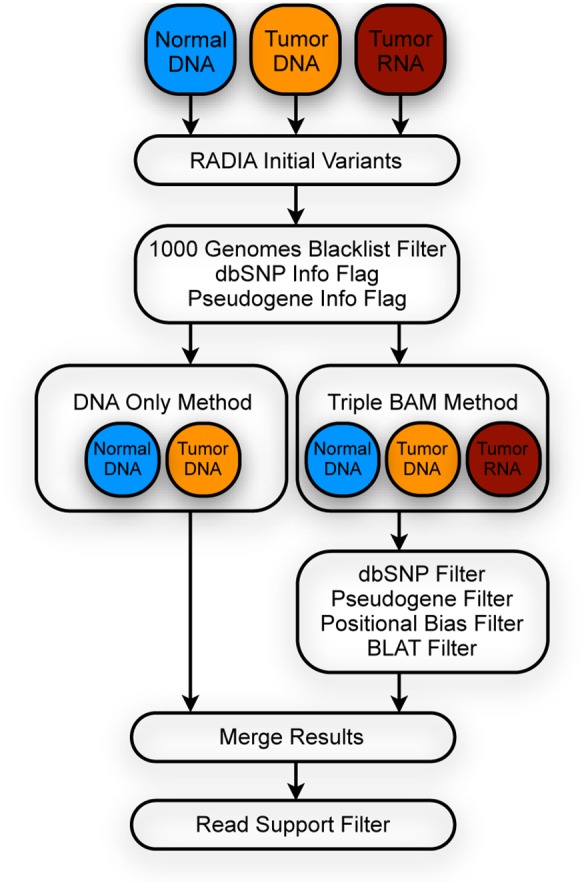
Overview of the RADIA work-flow for identifying somatic mutations. The normal DNA, tumor DNA, and tumor RNA BAMs are processed in parallel and initial low-level variants are identified. The variants are filtered by the DNA Only Method using the pairs of normal and tumor DNA and by the Triple BAM Method using all three datasets. The mutations from the two methods are merged and output in VCF format.

RADIA combines patient-matched tumor and normal DNA with the tumor RNA to detect somatic mutations. The DNA Only Method (DOM) (Figure 1) uses just the tumor/normal pairs of DNA (ignoring the RNA), while the Triple BAM Method (TBM) (Figure 1) uses all three datasets from the same patient to detect somatic mutations. The mutations from the TBM are further categorized into two sub-groups: RNA Confirmation and RNA Rescue mutations (Figure S1). RNA Confirmation mutations are those that are made by both the DOM and the TBM due to the strong variant read support in both the DNA and RNA. RNA Rescue mutations are those that had very little DNA support, hence not called by the DOM, but strong RNA support, and thus called by the TBM. RNA Rescue mutations are typically missed by traditional methods that only interrogate the DNA.

We have applied RADIA to data derived from over 3,300 patients representing 15 different cancer types from TCGA (Table S1). Overall, the RNA Rescue mutations that are made possible by the incorporation of the RNA-Seq data provide a two to seven percent increase in somatic mutations compared to the DOM (Table S1). Many of these mutations were new discoveries that were not previously found by other mutation calling algorithms in TCGA. Of these new discoveries, some mutations were found in well-known cancer genes that were heavily mutated in a specific cohort. We also find mutations in new samples where the same gene has already been identified as harboring mutations in other samples from the cohort. When these RNA Rescue mutations are added to the DNA Only mutations, these genes achieve a statistically significant overall mutation rate for the cohort.

Here we specifically focus on results from 177 endometrial carcinoma [13] and 230 lung adenocarcinoma [14] patients from TCGA. To demonstrate the increase in sensitivity from including the tumor RNA-Seq dataset, we simulated mutations by spiking them into the tumor DNA and tumor RNA of a breast cancer patient using bamsurgeon (https://github.com/adamewing/bamsurgeon). We also evaluated sensitivity and precision on the endometrial carcinoma and lung adenocarcinoma data using validation data that was generated by TCGA. We highlight RNA Rescue mutations found by the TBM in tumor suppressor genes such as *TP53*, *STK11*, and *CDKN2A* in lung adenocarcinoma.

## Methods

RADIA operates on two or more BAM files, producing somatic mutation calls through a series of steps outlined in Figure 1. Each step in this process is described in detail, beginning with the initial selection of sites for further processing and ending with a description of filters used to eliminate false positives while maintaining true positives.

### 2.1 Variant Detection with RADIA

RADIA is typically run on three BAM [5] files consisting of a pair of patient-matched tumor and normal genomes and a tumor transcriptome and outputs germline (inherited) variants and somatic Single Nucleotide Variants (SNVs). Here we focus specifically on the detection of somatic SNVs with RADIA. The DOM is run on the pairs of tumor and matched-normal DNA while the TBM is applied to the DNA and RNA triplets. After the DOM and TBM specific filters, the results are merged and run through a final read support filter (Figure 1). If RNA-Seq data is not available, RADIA can utilize paired tumor and normal DNA genomes using the DOM to detect germline variants and somatic SNVs.

Internally, RADIA uses the samtools [5] mpileup command (version 0.1.18) to examine the pileups of bases in each sample in parallel. A heuristic algorithm determines the existence and type of variant at any given position based on the user-configurable minimum thresholds for overall depth, variant depth, Base Alignment Quality (BAQ) [15], and mapping quality. Initially, RADIA requires a minimum overall depth of four bases, minimum variant depth of two bases, minimum phred BAQ of 10, and minimum phred mapping quality of 10. These initial calls are lenient in coverage and provide a good baseline set of calls for further filtering.

RADIA scans pileups of reads across the reference genome and outputs variants in Variant Call Format (VCF) (https://github.com/samtools/hts-specs). For each position, summary information such as the overall depth, allele specific depth and frequency, average BAQ base quality, average mapping quality, and the fraction of reads on the plus strand are calculated for both the DNA and RNA. All of this information is used during the filtering process.

### 2.2 Variant Filtering

After the initial variants are detected, a number of filters are applied to remove false positive variants that result from biological and technical artifacts. Each filter is described here in detail.

#### 2.2.1 Filtering Around INDELs

Many current mutation calling algorithms have a pre-processing step to account for misaligned reads around INDELs. This realignment step is computationally expensive and relies on accurately predicting the location of INDELs which itself is not a trivial problem. Base Alignment Quality (BAQ) is an alternative option for dealing with alignment ambiguity around INDELs. It calculates the probability that a base has been misaligned and returns the minimum of the original base quality and the base alignment quality. BAQ is run by default when executing a samtools mpileup command and has been shown to improve SNP calling accuracy [15]. We use the extended version of BAQ (option –E) that is activated by default in the latest version of samtools (0.1.19) for increased sensitivity and slightly lower specificity [5].

#### 2.2.2 1000 Genomes Blacklist Filter

The 1000 Genomes Project coined the term “accessible genome” to be the part of the reference genome that is reliable for accurate variant calling after removing ambiguous or highly repetitive regions [16]. Since the reference genome is incomplete, repetitive in places, and does not represent human genetic variation comprehensively, reads often get mapped incorrectly in locations outside the accessible genome (inaccessible sites), leading to false positive variant calls. Over 97% of inaccessible sites are due to high copy repeats or segmental duplications. In the pilot, the 1000 Genomes Project determined that 85% of the reference sequence and 93% of the coding region was accessible. Due to longer read lengths (75–100 bp) and improvements to both paired end protocols and sequence alignment algorithms, the accessible genome increased in Phase I to 94% of the reference and 98% of the coding region [17]. We filter variants that are not in the accessible genome using the Phase I mapping quality and depth blacklists (ftp://ftp-trace.ncbi.nih.gov/1000genomes/ftp/phase1/analysis_results/supporting/accessible_genome_masks/).

#### 2.2.3 Strand-Bias Filter

It has recently been shown that variant allele reads that occur exclusively on one strand are largely associated with false positives [8]. In order to account for this technical artifact, we filter based on the variant allele strand bias. If we have at least four total reads supporting the variant allele, then we apply the strand bias filter if more than 90% of the reads are on the forward strand or more than 90% are on the reverse strand.

#### 2.2.4 Filtering by mpileup Support

RADIA can be executed on patient-matched pairs of tumor and normal DNA samples using the DOM to identify germline variants and somatic mutations. We first compare the matched normal DNA to the human reference genome. We require the normal DNA to pass the mpileup support filters described in Table 1 for all germline variants.

**Table 1:** DNA Only Method mpileup Support Filters.

**Filter**	**Germline**	**Somatic**	
	**Normal DNA**	**Normal DNA**	**Tumor DNA**
Min Total Depth	10	10	10
Min Alt. Depth	4	NA	4
Min Alt. Percent	10%	NA	10%
Min Avg. Alt. BAQ	20	NA	20
Max Alt. Strand Bias	90%	NA	90%
Max Alt. Percent	NA	2%	NA
Max Other Percent	2%	2%	2%

The germline variants and somatic mutations from the DOM are filtered according to the parameters described here. The minimum average alternative read BAQ filter uses the phred scale. The maximum other percent restricts the percentage of reads that are allowed to support an additional alternative allele.

If no germline variant is found, we compare the tumor DNA to the matched normal DNA and the reference genome to search for somatic mutations. We require the normal DNA and tumor DNA to pass the mpileup support filters shown in Table 1 for all somatic variants. To ensure that we have the power to detect a possible germline variant at this site, we require that the germline DNA depth is 10 or more.

We use the Triple BAM Method to augment our somatic mutation calls using both the pairs of DNA and the RNA-Seq data. The normal DNA, tumor DNA, and tumor RNA must pass the mpileup support filters shown in Table 2 for all somatic mutations. We require at least one read with a minimum BAQ phred score of 15 in the tumor DNA. To rule out possible germline variants, we again require that the normal DNA depth is 10 or more. In addition, we filter out calls that overlap with common SNPs that are not flagged as clinically relevant and found in at least one percent of the samples in dbSNP [18]. We downloaded this subset of dbSNP from the “Common SNPs” track on the UCSC human genome browser [19], [20]. We found that many false positive variants overlapped with earlier versions of dbSNP. These variants were due to technical artifacts and were removed from subsequent versions of dbSNP [21]. Therefore, we filter out all variants that overlap with dbSNP versions 130, 132 or 135 (ftp://ftp.ncbi.nih.gov/snp/). The TBM calls are subjected to further filtering procedures as shown in Figure 1 and described below.

**Table 2:** Triple BAM mpileup Support Filters.

Filter	Somatic		
	Normal DNA	Tumor DNA	Tumor RNA
Min Total Depth	10	1	10
Min Alt. Depth	NA	1	4
Min Alt. Percent	NA	NA	10%
Min Avg. Alt. BAQ	NA	15	15
Max Alt. Strand Bias	NA	90%	90%
Max Alt. Percent	10%	NA	NA
Max Other Percent	10%	10%	2%

The somatic mutations from the TBM are filtered according to the parameters shown here.

#### 2.2.5 Pseudogene Filter

We noticed that many of our TBM mutations overlapped with predicted pseudogenes. Although expressed pseudogenes have recently been reported to be significant contributors to the transcriptional landscape and shown to play a role in cancer progression [22], mutations that overlap with predicted pseudogenes have a high false positive rate. Sequence similarity of pseudogene copies to their parent genes leads to uncertainty in alignment within these regions. Because of these technical artifacts, we remove TBM mutations that overlap with pseudogenes annotated in GENCODE by the ENCODE project (version 19) [23] and predicted by RetroFinder (version 5) [23], [24]. We downloaded the pseudogene annotations from the following tracks on the UCSC human genome browser [19], [25]: Gene Annotations from ENCODE/GENCODE and Retroposed Genes. The predicted pseudogenes occupy 1.5% of the total genome.

#### 2.2.6 Highly Variable Genes Filter

We remove TBM mutations that overlap with families of genes that have high sequence similarity. Some examples of these gene families are Human Leukocyte Antigens (HLAs), Ribosomal Proteins (RPLs), and immunoglobulins. While mutations in these genes may exist, special processing would be needed to distinguish them from false positive calls due to misaligned reads. We annotate the mutations using SnpEff [26] and filter out the following five gene families: RPLs, RP11s, HLAs, IGHVs and IGHCs.

#### 2.2.7 Positional Bias Filter

False positive calls are associated with misaligned reads where the alternative allele is consistently within a certain distance from the start or end of the read. The positional bias filter is applied when 95% or more of the reads that have an alternative allele are such that the alternate allele falls in the first third or last third of the read.

#### 2.2.8 BLAT Filter

We observed multiple instances where RNA-Seq reads appeared to be incorrectly mapped due to the added difficulties in aligning RNA-Seq data, such as dealing with hard to identify splice junctions and multiple gene isoforms. To guarantee that the RNA-Seq reads that support a variant do not map better to another location in the genome, we created a BLAT filter. All of the RNA-Seq reads that support a variant are extracted from the BAM file and aligned to the human genome using BLAT [27]. If the read maps to another location with a better score, the read is rejected. After using BLAT on each read, we again require that there are at least four valid reads that support the variant and that 10% or more of the reads support the variant.

#### 2.2.9 Read Support Filter

We merge the calls from the DOM and the TBM and apply one final filter. We require that each somatic mutation be supported by at least four “perfect” reads. We define a perfect read as follows:

1. Minimum mapping quality of read is 10
2. Minimum base quality of alternative allele in read is 10
3. Minimum base qualities of the five bases up- and down-stream of the alternative allele are 10
4. Read is properly paired
5. Read has fewer than four mismatches across its entirety when compared to the reference
6. Read does not require an insertion or deletion to be mapped

After determining the number of perfect reads that support the reference and the alternative at a coordinate, we re-apply the strand bias filter to guarantee that no more than 90% of the total perfect reads are from one strand.

## Results

We evaluate the sensitivity of RADIA using simulation data that was generated from patient data. We also measure the sensitivity and precision of RADIA using patient and validation data generated by TCGA. All patients in this study provided written informed consent to genomic studies in accordance with local Institutional Review Boards (Table S2) and the policies and guidelines outlined by the Ethics, Law and Policy Group from TCGA. All patient data is anonymous and was originally collected for routine therapeutic purposes.

### 3.1 Sensitivity on Simulation Data

In order to evaluate sensitivity and demonstrate the increase in power from including the RNA-Seq data, we simulated somatic mutations starting from patient data. We spiked mutations into a pair of breast cancer tumor DNA and tumor RNA samples using bamsurgeon (https://github.com/adamewing/bamsurgeon), a tool we developed to generate simulation data that closely mimics actual experimental data from high-throughput sequencing datasets. Bamsurgeon first determines the loci that have an appropriate DNA and RNA depth to spike in mutations. It then extracts the reads at the loci, adjusts the VAF according to the user-defined VAF distribution, and then re-maps the reads (Figure S2). This simulation strategy is more sophisticated than simply generating simulated reads from a reference genome, as it retains the biological and technical artifacts that are inherently present in next generation sequencing data. We performed two spike-in experiments: one varying the DNA VAF while holding the RNA VAF constant, and one varying the RNA VAF while holding the DNA VAF to 10% or less.

#### 3.1.1 Sensitivity on Variable DNA-Constant RNA Simulation Data

**Figure 2:**
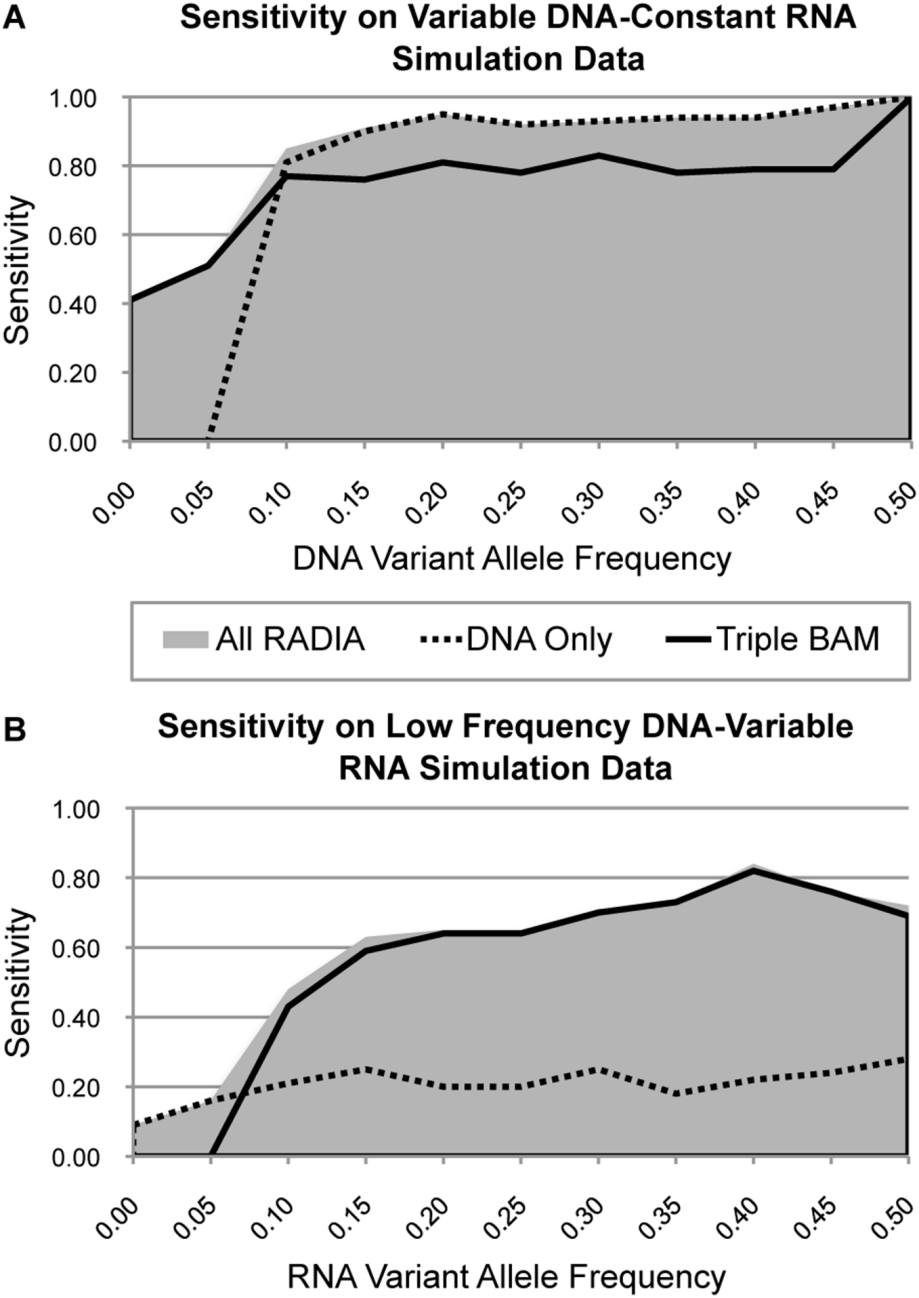
Sensitivity of RADIA on simulation data. Artificial mutations were spiked into the tumor DNA and RNA BAM files of a breast cancer patient using bamsurgeon. (A) Mutations were spiked into the DNA at variant allele frequencies distributed from 1–50% and into the RNA at a constant 25%. The overall sensitivity of RADIA was 85%. RNA Rescue calls from the Triple BAM method detected the mutations that had a DNA VAF less than 10%. (B) Mutations were spiked into the DNA at 10% or less and into the RNA distributed from 1–50%. Most of the DOM mutations are filtered due to the low DNA allelic frequency. The mutations that have adequate RNA read support are rescued back at these low DNA allelic frequencies.

To evaluate the sensitivity of RADIA, we spiked in 1,594 mutations to the tumor DNA sequence with a variant allele frequency ranging from 1–50% and to the tumor RNA sequence at a constant frequency of 25%. The overall sensitivity rate averaged across all VAFs is 85% consisting of 1,351 out of 1,594 spiked in mutations (Figure 2A). Of the 243 calls that were filtered out, over 50% are removed because they failed to meet the minimum variant allele frequency, more than 20% land in blacklist regions that the method ignores, and nearly 20% are discarded due to the BLAT filter. The number of mutations that are rejected by the full list of filters can be found in Figure S3.

#### 3.1.2 Sensitivity on Low Frequency DNA-Variable RNA Simulation Data

To demonstrate the ability of the TBM to rescue calls at low DNA VAFs, we spiked in 1,761 mutations to the tumor RNA sequence with a variant allele frequency ranging from 1–50% and to the tumor DNA sequence at a frequency of 10% or less. Most of the mutations by the DOM are filtered out due to the low allelic frequency in the DNA (Figure S4). For the mutations that have sufficient read support in the RNA, these low DNA VAFs are rescued back (Figure 2B).

### 3.2 Precision and Sensitivity on Patient Data

We made somatic mutation calls on 177 non-hypermutated TCGA endometrial carcinoma samples [13]. All 177 tumor and matched normal whole exome sequencing and RNA-Seq alignments in BAM [5] format were downloaded from TCGA at the Cancer Genomics Hub (CGHub, https://cghub.ucsc.edu, Table S2). The exomes were sequenced using the Illumina Genome Analyzer II, and the paired-end sequencing reads were aligned by BWA [28]. The RNA was sequenced using the Illumina Genome Analyzer II, and the single-end sequencing reads were aligned by MapSplice (V2) [29].

#### 3.2.1 RADIA Precision on Endometrial Carcinoma Patient Data

For the study on endometrial carcinoma by TCGA [13], mutations were submitted by three independent TCGA Genomic Data Analysis Centers (GDACs). These mutations were merged and targeted for custom recapture and resequencing using new cDNA libraries from the tumor and normal DNA samples [13]. We downloaded the validation BAMs containing the results of the hybrid capture and resequencing of targeted mutations from CGHub (https://cghub.ucsc.edu, Table S2). We utilized the identical validation criteria used by the TCGA Endometrial Analysis Working Group to validate the somatic mutations detected by RADIA [13]. For each somatic mutation, we queried the patient-matched tumor and normal validation data. We required at least 10 reads in both the tumor and normal data in order to determine if a call validated, otherwise we classified it as ambiguous. If the variant was present at low levels in both datasets, we also classified it as ambiguous. Otherwise, we determined whether a mutation validated as germline/LOH, somatic, or neither according to Table 3. In addition, any RNA Rescue call in the “Not Validated” group that overlapped with a COSMIC somatic mutation that was confirmed in another study was considered as validated.

**Table 3:** Validation Criteria for Endometrial Carcinoma Data.

Normal VAF	Tumor VAF			
	0%	<8%	≥8%, <20%	≥20%
= 0%	*Not Validated*	**Somatic Low**	**Somatic Med**	**Somatic High**
<3%	*Not Validated*	Ambiguous	**Somatic Med**	**Somatic High**
≥3%	*Germline/LOH*	*Germline/LOH*	*Germline/LOH*	*Germline/LOH*

Validation BAMs were used to determine the validation status for somatic mutations as shown here. A mutation is considered validated in the Somatic Low, Med, or High groups (bold), not validated in the “Not Validated” (italics) and Germline/LOH groups (italics), and Ambiguous when there was low read depth (<10 reads) or low VAFs in both the normal (<3%) and tumor (<8%) validation BAMs.

We made a total of 27,900 somatic mutation calls over 177 endometrial samples, of which the DOM and TBM made 27,390 and 6,325 calls respectively. Of the 6,325 TBM calls, there were 5,815 RNA Confirmation mutations that were made by both the DOM and TBM signifying high DNA and RNA support, and importantly, a total of 510 RNA Rescue mutations that were missed by the DOM.

**Figure 3:**
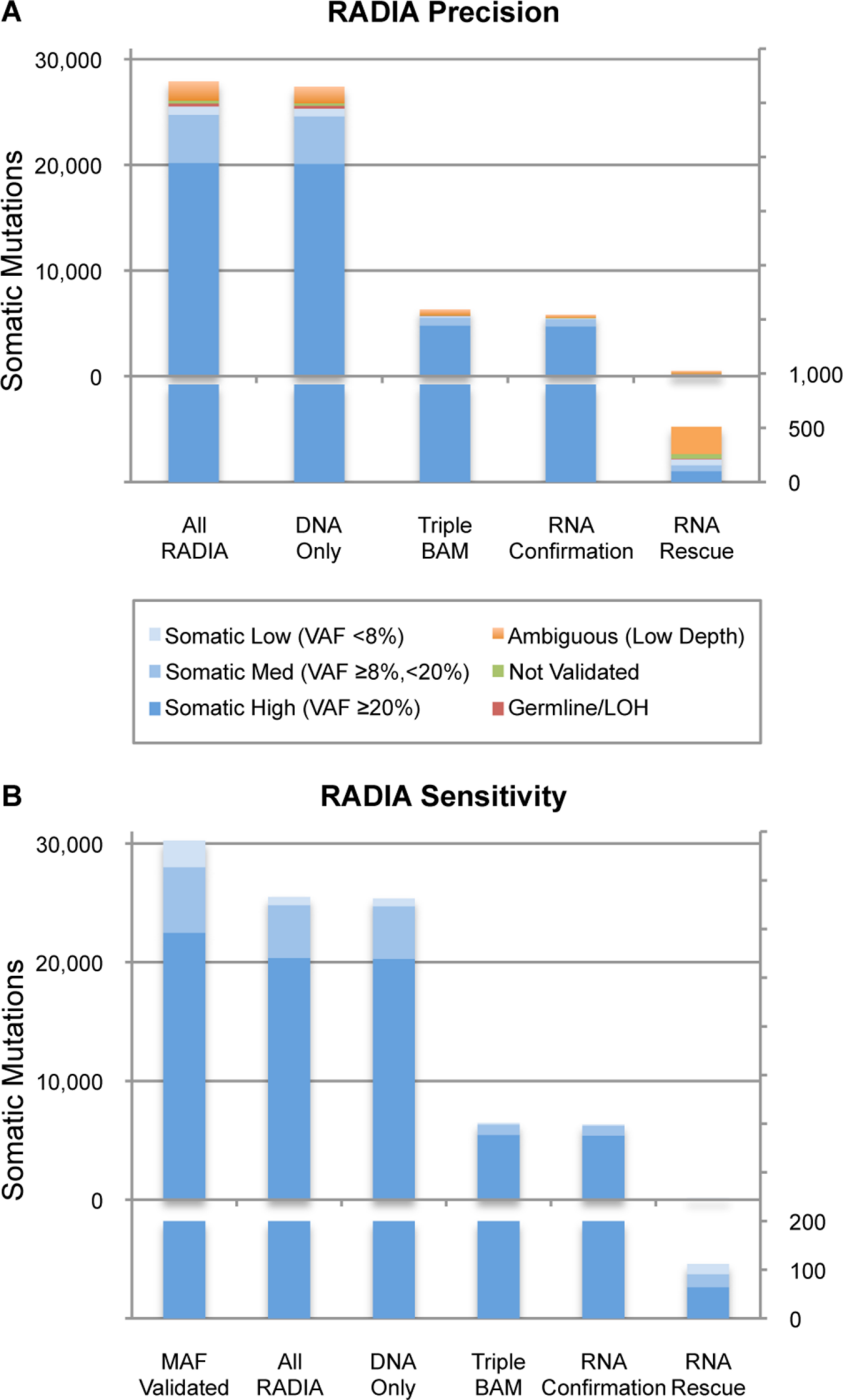
Precision and sensitivity of RADIA on 177 non-hypermutated endometrial carcinoma samples. Mutations are considered validated in the Somatic Low, Med, or High groups (blue), not validated in the “Not Validated” (green) and Germline/LOH (red) groups, and Ambiguous (orange) when there was low read depth (<10 reads) or ambiguity in the validation data. (A) An overall precision of 98% was demonstrated. RNA Confirmation mutations with strong DNA and RNA support validated over 99%. RNA Rescue mutations validated at 74%. (B) The union of all mutations submitted by TCGA GDACs that validated as somatic was considered as the truth set. RADIA demonstrated an overall sensitivity rate of 84%. Of the mutations that were missed, 33% occurred at low variant allele frequencies (<8%) and 23% occurred in blacklist regions that were ignored.

Using the validation strategy described above, we demonstrate that the overall precision for RADIA is 98% (Figure 3A). Due to lack of coverage or uncertainty in the tumor and normal validation BAMs, a total of 1,825 calls were considered to be ambiguous. Of the remaining 26,075 mutations called by RADIA, 25,520 validated as somatic, 271 validated as germline/LOH variants and 284 did not validate. The precision of calls made by the DOM and the TBM was 98% and 98.5% respectively. For the RNA Confirmation mutations made by both the DOM and the TBM, the precision was 99.3%. There were 510 RNA Rescue mutations made only by the TBM, and even though most of these calls were not targeted for validation, the precision was 74%. For the 510 RNA Rescue calls, 251 were classified as ambiguous, 6 validated as Germline/LOH, and 61 did not validate. Of the remaining 192 RNA Rescue mutations that validated, 178 (93%) were verified using the validation BAMs and 14 (7%) were confirmed as somatic mutations in COSMIC.

We next examined the precision of the DOM with varying RNA-Seq reads supporting the variant allele as well as the precision of RNA Rescue mutations with differing levels of DNA supporting reads. Sixty-two percent of the DOM mutations were covered by reads in the RNA-Seq data, and 29% had at least 10 RNA-Seq reads covering the mutation. Nearly half (44%) had at least one RNA read supporting the DNA variant allele, while 25% of the DOM mutations had at least four supporting RNA reads. The precision of the DOM is lowest (92%) with no RNA-Seq support, increases to 95% with weak RNA-Seq support (at least one but less than five supporting reads), and increases to 99.3% for RNA Confirmation mutations. Overall, mutations that are detected by the DOM validate above 92%, regardless of the RNA-Seq support, and the precision increases as the RNA-Seq support increases.

On the other hand, RNA Rescue mutations weakly supported by the DNA validate at low levels. For RNA Rescue mutations, we require at least one variant supporting read in the DNA in order to distinguish between RNA Rescue mutations and possible RNA editing events. The precision of RNA Rescue mutations with only one read supporting the variant in the DNA was 11%, with two supporting reads in the DNA 23%, with three supporting reads in the DNA 43%, and with four or more supporting reads in the DNA 94%.

#### 3.2.2 RADIA Sensitivity on Endometrial Carcinoma Patient Data

In order to measure the sensitivity of RADIA, we considered the union of all mutations submitted by TCGA GDACs that validated as somatic as our truth set. There were 30,239 mutations that validated as somatic from TCGA. We compared our somatic mutations to this truth set and demonstrated an overall sensitivity of 84% (Figure 3B, Figure S5). Of the 4,751 calls that were missed, 1,539 (33%) were filtered by RADIA because they had a variant allele frequency less than 8% (Figure S6). In addition, 1,072 (23%) landed in blacklist regions that were not considered (Figure S6).

#### 3.2.3 RADIA Precision on Lung Adenocarcinoma Patient Data

Finally, RADIA somatic mutations were analyzed during the course of our participation in the TCGA Lung Adenocarcinoma Analysis Working Group [14]. We ran RADIA on 230 TCGA lung adenocarcinoma triplets that we downloaded from CGHub (https://cghub.ucsc.edu, Table S2). The exomes were sequenced using the Illumina HiSeq platform, and the paired-end sequencing reads were aligned by BWA [28]. The RNA was sequenced using the Illumina HiSeq platform, and the paired-end sequencing reads were aligned by MapSplice (V2) [29]. Validation was performed by the Broad Institute on 74 genes of interest along with an additional 1,150 somatic SNVs. Validation was attempted on 2,404 RADIA somatic mutations and 2,395 (99.63%) were verified. From the DOM, 2,336 of the 2,345 mutations (99.62%) validated. Importantly, 469/469 (100%) of the TBM mutations consisting of 410 RNA Confirmation and 59 RNA Rescue mutations validated.

### 3.3 Somatic Mutations in Specific Lung Adenocarcinoma Genes

Mutations in the tumor suppressor gene *TP53* are common in the majority of human cancers. Most of the mutations occur in the DNA-Binding Domain (DBD) and are considered change-of-function mutations that alter activity of *TP53*, sometimes acting in a dominant negative manner to sequester wildtype tp53 protein *in trans* [30]. As such, many p53 mutant proteins endow cells with oncogenic characteristics by promoting cell proliferation, survival, and metastasis [31].

We ran RADIA on 230 TCGA lung adenocarcinoma triplets [14] and discovered two non-synonymous *TP53* mutations that were below the detection threshold for other mutation calling algorithms used by TCGA (Table 4). Both of the mutations were validated by the deep-sequencing validation data and confirmed as somatic in COSMIC by other studies. One of the mutations (G266E) was confirmed as somatic in another lung cancer study [32] as well as in prostate [33], pancreas [34], urinary tract [35], and hematopoietic and lymphoid [36] cancer studies. The G266E mutation occurs in the *TP53* DBD mutation hotspot frequently resulting in pathological effects [37]–[39]. This mutation has also been described as a gain-of-function mutation in a melanoma cell line [40]. The other *TP53* mutation (G199V) was confirmed as somatic in breast [41], ovarian [42], and medulloblastoma [43] studies. It is a known anti-apoptotic gain-of-function mutation that promotes cell survival through the signal transducer and activator of transcription-3 (STAT3) pathway [44]. Knockdown experiments of G199V p53 mutants demonstrated a level of anti-tumor activity similar to high doses of chemotherapeutic agents, suggesting that inhibition of G199V p53 mutants may be beneficial for cancer treatment [44].

**Table 4:** RNA Rescue Mutations in Lung Adenocarcinoma not Detected by Other Methods in TCGA.

Gene	Mutation	DNA VAF	RNA VAF	Validation DNA VAF
*TP53*	G266E	1/7 (13%)	6/10 (60%)	47/183 (26%)
*TP53*	G199V	4/64 (6%)	8/57 (14%)	17/380 (4%)
*CDKN2A*	R131H	3/45 (7%)	22/62 (35%)	9/149 (6%)
*CDKN2A*	R122*/R163*	2/16 (13%)	31/34 (91%)	20/92 (22%)
*STK11*	W239*	1/13 (7%)	20/40 (50%)	NA

These mutations were below the detection threshold for other mutation calling algorithms used by TCGA. The ratio of reads supporting the mutations along with the variant allele frequencies are shown for both the DNA and RNA. Validation was done on four of the mutations, and the resulting validation DNA variant allele frequencies are shown.

Additionally, we found mutations in other well-known tumor suppressor genes such as *STK11* and *CDKN2A*. In the lung adenocarcinoma manuscript from TCGA, mutations in *STK11* and *CDKN2A* were reported in 17% and 4% of all patients, respectively [14]. *STK11* was the fourth most mutated gene and *CDKN2A* was the sixteenth [14]. The proximal-proliferative subtype in lung adenocarcinoma is characterized by an enrichment of mutations in *KRAS* along with inactivation mutations in *STK11* [14]. In the *STK11* gene, we discovered a nonsense mutation at W239* in the structurally conserved protein kinase domain that was below the detection threshold for other mutation algorithms used by TCGA. This mutation introduces an early stop codon in exon five (of ten) leading to a truncated protein. This site is in COSMIC and was previously reported to be part of a 398 nucleotide deletion in a lung cancer study [45].

In the *CDKN2A* gene, we found one nonsense mutation at R122*, R163* and one missense mutation at R131H, R80H that were both validated by TCGA and found in COSMIC. *CDKN2A* is silenced in many CpG island methylator phenotype-high (CIMP-High) tumors by DNA methylation [14], but mutations and deletions in *CDKN2A* also result in loss of function. The nonsense mutation at R122*, R163* results in an early stop codon in exon two (of three or four, isoform dependent) leading to a truncated protein. Previous lung cancer studies [46]–[48] have reported frameshifts and deletions at this site. The missense mutation at R131H was also found in colon cancer [49], clear cell sarcoma [50], and chronic myeloid leukemia [51] and confirmed as somatic in biliary tract cancer [52].

## Discussion

Identifying somatic mutations is a key step in characterizing the cancer genome. Until now, algorithms for mutation detection have concentrated on comparing just the normal and tumor genomes within the same individual. In the past few years, it has become common to also sequence the tumor transcriptome using RNA-Seq technologies. Large genomics studies, such as those conducted by TCGA, primarily use the RNA-Seq for gene expression, gene fusion, and splicing analyses. With the cost of sequencing steadily decreasing and the wealth of information that can by obtained from RNA-Seq, we predict that the sequencing of the tumor RNA will continue to be routine in large cancer profiling projects. We have developed a novel method called RADIA that combines the normal DNA, tumor DNA, and tumor RNA from the same individual to increase sensitivity to detect somatic mutations without compromising specificity. Here we have focused on the ability of RADIA to detect germline variants and somatic single nucleotide variants. In the future, we plan to include other classes of somatic mutations such as small insertions and deletions (INDELs), loss of heterozygosity events (LOHs) and RNA editing events.

The accurate detection of somatic mutations is complicated by biological and technical artifacts such as tumor purity and subclonality, varying allele frequencies, sequencing depths, and copy-number variation. There is a trade-off between high sensitivity and high specificity, such that it is difficult to achieve both. By including an additional dataset, we are increasing our ability to reliably detect mutations, especially at low variant allele frequencies (Figure S7) where the signal to noise ratio becomes unfavorable.

Many widely used mutation calling algorithms see a large decrease in precision as the DNA variant allele frequency declines [6], [8], [9], [11], [12]. We found that a DNA VAF of 10% gives us the best balance between sensitivity and precision. To demonstrate this point, we lowered the DNA VAF to 5% and reran RADIA on the endometrial carcinoma data from Section 3.2. We used the same validation strategy as described in Section 3.2 and compared the results to the ones with a DNA VAF of 10%. We found a slight 1% increase in overall sensitivity from 84% (at 10% VAF) to 85% (at 5% VAF) but an 8% decrease in overall precision from 97% (at 10% VAF) to 89% (at 5% VAF).

By combining the RNA with the DNA, we are able to confirm the expression of a mutation, providing insight into its likely functional effect. Confirming mutations through RNA-Seq is also advantageous for large genomic studies in providing a means for weak validation for mutations without costly resequencing for validation (Figure S8). We find that over 99% of mutations that have both strong DNA and RNA support validate upon resequencing, suggesting that if one is not using mutations in clinical practice but rather estimating overall frequencies of specific mutations in a research cohort, the extreme expense in validating every mutation may not be warranted. While the integration of RNA and DNA provides an important but limited use as a DNA variant validation technique, studying the impacts on gene expression levels may lead to a deeper understanding of the functional impact of DNA-originating variants.

Here we have outlined some of the strengths of RADIA, but approaches that use RNA-Seq for detecting variants have clear limitations [53], [54]. Only expressed alleles can be evaluated, which reduces the number of genes that can be assessed. In addition, several classes of mutations, such as the introduction of premature stop codons that lead to nonsense mediated decay, cannot be verified. Expression levels can also confound the ability to detect an imbalance in the genomic VAF as influences due to feedback control to rebalance gene dosage are currently unknown.

With RADIA, we are able to detect mutations in important cancer genes such as *TP53* that were previously not identified by other algorithms because the signal was lost in the noise. Somatic mutations are commonly used to group patients into subtypes that are critical for diagnosis and treatment of the disease. Our ability to rescue back mutations for individual patients will assist in correctly identifying each patient’s specific subtype and consequently their treatment options.

## Supporting Information

Figure S1 **Schematic of mutations detected by the DNA Only Method (DOM) and Triple BAM Method (TBM).** In the first and middle columns, there is enough DNA read support for the DOM and other algorithms acting on DNA pairs to detect a mutation. In the middle and last columns, there is sufficient RNA read support for the TBM to detect a mutation. The middle column illustrates “RNA Confirmation” mutations that are detected by both the DOM and the TBM due to high read support in both the DNA and RNA. The last column represents the “RNA Rescue” mutations that have some support in the DNA and strong evidence in the RNA. The RNA Rescue mutations are typically missed by traditional mutation calling algorithms that only investigate the pairs of DNA.
(PDF)

Figure S2 **Diagram of bamsurgeon methodology.** Mutations are spiked into BAM files by selecting locations with adequate coverage, extracting the reads, and adjusting the VAF according to the desirable VAF distribution. Once the bases in the reads are changed, the reads are remapped to the genome, replacing the reads in the original BAM file.
(PDF)

Figure S3 **Filters applied in the Variable DNA-Constant RNA bamsurgeon simulation experiment.** The DNA variant allele frequencies were distributed from 1–50% and the RNA was held constant at 25%. Most of the DOM mutations were filtered because of the low variant allele frequency and tumor strand bias. In the TBM, most of the mutations were filtered due to the minimum number of alternative alleles required to make a call (n = 4) and strand bias in the tumor DNA and RNA.
(PDF)

Figure S4 **Filters applied in the Low Frequency DNA-Variable RNA bamsurgeon simulation experiment.** The RNA variant allele frequencies were distributed from 1–50% and the DNA was held at 10% or less. Most of the DOM mutations were filtered because of the low DNA variant allele frequency and tumor strand bias. In the TBM, most of the mutations were filtered due to the minimum number of alternative alleles required to make a call (n = 4) and the low RNA variant allele frequency.
(PDF)

Figure S5 **Distribution of overlaps between RADIA and the endometrial TCGA MAF file.** The distribution of the overlaps between RADIA and the validated somatic mutations from the endometrial TCGA network MAF file.
(PDF)

Figure S6 **Filters applied to the RADIA mutations that validated as somatic in the endometrial TCGA MAF file.** Thirty-three percent of the mutations had a DNA VAF of eight percent or less while 23% landed in blacklist regions that were ignored.
(PDF)

Figure S7 **RNA Rescue mutations are primarily at low DNA VAFs.** RNA Rescue mutations are primarily found at low DNA variant allele frequencies, but they also occur at higher frequencies where they were filtered due to non-depth related artifacts (e.g. strand-bias).
(PDF)

Figure S8 **Distribution of RNA Confirmation Calls.** The total number of mutations (blue) that are covered by at least one RNA read (yellow), one RNA read supporting the alternative allele (orange), and RNA Confirmation mutations with high support in both the DNA and RNA (purple).
(PDF)

Table S1 **Summary of TCGA samples analyzed by RADIA.** RADIA has been run on over 3,300 TCGA samples across 15 different types of cancer. The RNA Rescue mutations make up two to seven percent of the total somatic mutations across the 15 types of cancer. Variant Call Format (VCF) and Mutation Annotation Format (MAF) files can be downloaded from the TCGA Data Portal (https://tcga-data.nci.nih.gov/tcga/). Open-access somatic MAFs can be visualized and downloaded via the UCSC Cancer Browser (https://genome-cancer.ucsc.edu/).
(PDF)

Table S2 **TCGA barcodes and Universally Unique Identifiers (UUIDs) for the TCGA samples used in this study.** All patients provided written informed consent in accordance with TCGA guidelines and local Institutional Review Boards (IRBs).
(XLSX)

## References

[1] Hanahan D, Weinberg RA (2000) The hallmarks of cancer. Cell 100: 57–70. 10647931 10.1016/s0092-8674(00)81683-9

[2] Hahn WC, Counter CM, Lundberg AS, Beijersbergen RL, Brooks MW, et al (1999) Creation of human tumour cells with defined genetic elements. Nature 400: 464–468. 10440377 10.1038/22780

[3] Wang Z, Gerstein M, Snyder M (2009) RNA-Seq: a revolutionary tool for transcriptomics. Nat Rev Genet 10: 57–63. 19015660 10.1038/nrg2484 PMC2949280

[4] Gott JM, Emeson RB (2000) Functions and mechanisms of RNA editing. Annu Rev Genet 34: 499–531. 11092837 10.1146/annurev.genet.34.1.499

[5] Li H, Handsaker B, Wysoker A, Fennell T, Ruan J, et al (2009) The Sequence Alignment/Map format and SAMtools. Bioinformatics 25: 2078–2079. 19505943 10.1093/bioinformatics/btp352 PMC2723002

[6] Koboldt DC, Zhang Q, Larson DE, Shen D, McLellan MD, et al (2012) VarScan 2: somatic mutation and copy number alteration discovery in cancer by exome sequencing. Genome Res 22: 568–576. 22300766 10.1101/gr.129684.111 PMC3290792

[7] Saunders CT, Wong WS, Swamy S, Becq J, Murray LJ, et al (2012) Strelka: accurate somatic small-variant calling from sequenced tumor-normal sample pairs. Bioinformatics 28: 1811–1817. 22581179 10.1093/bioinformatics/bts271

[8] Larson DE, Harris CC, Chen K, Koboldt DC, Abbott TE, et al (2012) SomaticSniper: identification of somatic point mutations in whole genome sequencing data. Bioinformatics 28: 311–317. 22155872 10.1093/bioinformatics/btr665 PMC3268238

[9] Koboldt DC, Chen K, Wylie T, Larson DE, McLellan MD, et al (2009) VarScan: variant detection in massively parallel sequencing of individual and pooled samples. Bioinformatics 25: 2283–2285. 19542151 10.1093/bioinformatics/btp373 PMC2734323

[10] Goya R, Sun MG, Morin RD, Leung G, Ha G, et al (2010) SNVMix: predicting single nucleotide variants from next-generation sequencing of tumors. Bioinformatics 26: 730–736. 20130035 10.1093/bioinformatics/btq040 PMC2832826

[11] Cibulskis K, Lawrence MS, Carter SL, Sivachenko A, Jaffe D, et al (2013) Sensitive detection of somatic point mutations in impure and heterogeneous cancer samples. Nat Biotechnol 31: 213–219. 23396013 10.1038/nbt.2514 PMC3833702

[12] Roberts ND, Kortschak RD, Parker WT, Schreiber AW, Branford S, et al (2013) A comparative analysis of algorithms for somatic SNV detection in cancer. Bioinformatics 29: 2223–2230. 23842810 10.1093/bioinformatics/btt375 PMC3753564

[13] Kandoth C, Schultz N, Cherniack AD, Akbani R, Liu Y, et al (2013) Integrated genomic characterization of endometrial carcinoma. Nature 497: 67–73. 23636398 10.1038/nature12113 PMC3704730

[14] The Cancer Genome Atlas (2014) Comprehensive molecular profiling of lung adenocarcinoma. Nature 511: 543–550. 25079552 10.1038/nature13385 PMC4231481

[15] Li H (2011) Improving SNP discovery by base alignment quality. Bioinformatics 27: 1157–1158. 21320865 10.1093/bioinformatics/btr076 PMC3072548

[16] 1000 Genomes Project Consortium (2010) A map of human genome variation from population-scale sequencing. Nature 467: 1061–1073. 20981092 10.1038/nature09534 PMC3042601

[17] 1000 Genomes Project Consortium (2012) An integrated map of genetic variation from 1,092 human genomes. Nature 491: 56–65. 23128226 10.1038/nature11632 PMC3498066

[18] Sherry ST, Ward MH, Kholodov M, Baker J, Phan L, et al (2001) dbSNP: the NCBI database of genetic variation. Nucleic Acids Res 29: 308–311. 11125122 10.1093/nar/29.1.308 PMC29783

[19] Kent WJ, Sugnet CW, Furey TS, Roskin KM, Pringle TH, et al (2002) The human genome browser at UCSC. Genome Res 12: 996–1006. 12045153 10.1101/gr.229102 PMC186604

[20] Karolchik D, Barber GP, Casper J, Clawson H, Cline MS, et al (2014) The UCSC Genome Browser database: 2014 update. Nucleic Acids Res 42: D764–770. 24270787 10.1093/nar/gkt1168 PMC3964947

[21] Musumeci L, Arthur JW, Cheung FS, Hoque A, Lippman S, et al (2010) Single nucleotide differences (SNDs) in the dbSNP database may lead to errors in genotyping and haplotyping studies. Hum Mutat 31: 67–73. 19877174 10.1002/humu.21137 PMC2797835

[22] Kalyana-Sundaram S, Kumar-Sinha C, Shankar S, Robinson DR, Wu YM, et al (2012) Expressed pseudogenes in the transcriptional landscape of human cancers. Cell 149: 1622–1634. 22726445 10.1016/j.cell.2012.04.041 PMC3597446

[23] Harrow J, Frankish A, Gonzalez JM, Tapanari E, Diekhans M, et al (2012) GENCODE: the reference human genome annotation for The ENCODE Project. Genome Res 22: 1760–1774. 22955987 10.1101/gr.135350.111 PMC3431492

[24] Baertsch R, Diekhans M, Kent WJ, Haussler D, Brosius J (2008) Retrocopy contributions to the evolution of the human genome. BMC Genomics 9: 466. 18842134 10.1186/1471-2164-9-466 PMC2584115

[25] Rosenbloom KR, Sloan CA, Malladi VS, Dreszer TR, Learned K, et al (2013) ENCODE data in the UCSC Genome Browser: year 5 update. Nucleic Acids Res 41: D56–63. 23193274 10.1093/nar/gks1172 PMC3531152

[26] Cingolani P, Platts A, Wang le L, Coon M, Nguyen T, et al (2012) A program for annotating and predicting the effects of single nucleotide polymorphisms, SnpEff: SNPs in the genome of Drosophila melanogaster strain w1118; iso-2; iso-3. Fly (Austin) 6: 80–92. 22728672 10.4161/fly.19695 PMC3679285

[27] Kent WJ (2002) BLAT–the BLAST-like alignment tool. Genome Res 12: 656–664. 11932250 10.1101/gr.229202 PMC187518

[28] Li H, Durbin R (2009) Fast and accurate short read alignment with Burrows-Wheeler transform. Bioinformatics 25: 1754–1760. 19451168 10.1093/bioinformatics/btp324 PMC2705234

[29] Wang K, Singh D, Zeng Z, Coleman SJ, Huang Y, et al (2010) MapSplice: accurate mapping of RNA-seq reads for splice junction discovery. Nucleic Acids Res 38: e178. 20802226 10.1093/nar/gkq622 PMC2952873

[30] Friedman PN, Chen X, Bargonetti J, Prives C (1993) The p53 protein is an unusually shaped tetramer that binds directly to DNA. Proc Natl Acad Sci U S A 90: 3319–3323. 8475074 10.1073/pnas.90.8.3319 PMC46291

[31] Muller PA, Vousden KH (2012) p53 mutations in cancer. Nat Cell Biol 15: 2–8. 10.1038/ncb2641 23263379

[32] Kan Z, Jaiswal BS, Stinson J, Janakiraman V, Bhatt D, et al (2010) Diverse somatic mutation patterns and pathway alterations in human cancers. Nature 466: 869–873. 20668451 10.1038/nature09208

[33] Lindberg J, Mills IG, Klevebring D, Liu W, Neiman M, et al (2013) The mitochondrial and autosomal mutation landscapes of prostate cancer. Eur Urol 63: 702–708. 23265383 10.1016/j.eururo.2012.11.053

[34] Biankin AV, Waddell N, Kassahn KS, Gingras MC, Muthuswamy LB, et al (2012) Pancreatic cancer genomes reveal aberrations in axon guidance pathway genes. Nature 491: 399–405. 23103869 10.1038/nature11547 PMC3530898

[35] Gui Y, Guo G, Huang Y, Hu X, Tang A, et al (2011) Frequent mutations of chromatin remodeling genes in transitional cell carcinoma of the bladder. Nat Genet 43: 875–878. 21822268 10.1038/ng.907 PMC5373841

[36] Abaan OD, Polley EC, Davis SR, Zhu YJ, Bilke S, et al (2013) The exomes of the NCI-60 panel: a genomic resource for cancer biology and systems pharmacology. Cancer Res 73: 4372–4382. 23856246 10.1158/0008-5472.CAN-12-3342 PMC4893961

[37] Pfaff E, Remke M, Sturm D, Benner A, Witt H, et al (2010) TP53 mutation is frequently associated with CTNNB1 mutation or MYCN amplification and is compatible with long-term survival in medulloblastoma. J Clin Oncol 28: 5188–5196. 21060032 10.1200/JCO.2010.31.1670

[38] Alsner J, Yilmaz M, Guldberg P, Hansen LL, Overgaard J (2000) Heterogeneity in the clinical phenotype of TP53 mutations in breast cancer patients. Clin Cancer Res 6: 3923–3931. 11051239 10.1186/bcr109 PMC3300808

[39] Fernandez-Cuesta L, Oakman C, Falagan-Lotsch P, Smoth KS, Quinaux E, et al (2012) Prognostic and predictive value of TP53 mutations in node-positive breast cancer patients treated with anthracycline- or anthracycline/taxane-based adjuvant therapy: results from the BIG 02–98 phase III trial. Breast Cancer Res 14: R70. 22551440 10.1186/bcr3179 PMC3446332

[40] Gartel AL, Feliciano C, Tyner AL (2003) A new method for determining the status of p53 in tumor cell lines of different origin. Oncol Res 13: 405–408. 12725531 10.3727/096504003108748429

[41] The Cancer Genome Atlas (2012) Comprehensive molecular portraits of human breast tumours. Nature 490: 61–70. 23000897 10.1038/nature11412 PMC3465532

[42] Jones S, Wang TL, Ie-Shih M, Mao TL, Nakayama K, et al (2010) Frequent mutations of chromatin remodeling gene ARID1A in ovarian clear cell carcinoma. Science 330: 228–231. 20826764 10.1126/science.1196333 PMC3076894

[43] Robinson G, Parker M, Kranenburg TA, Lu C, Chen X, et al (2012) Novel mutations target distinct subgroups of medulloblastoma. Nature 488: 43–48. 22722829 10.1038/nature11213 PMC3412905

[44] Kim TH, Lee SY, Rho JH, Jeong NY, Soung YH, et al (2009) Mutant p53 (G199V) gains antiapoptotic function through signal transducer and activator of transcription 3 in anaplastic thyroid cancer cells. Mol Cancer Res 7: 1645–1654. 19825993 10.1158/1541-7786.MCR-09-0117

[45] Davies H, Hunter C, Smith R, Stephens P, Greenman C, et al (2005) Somatic mutations of the protein kinase gene family in human lung cancer. Cancer Res 65: 7591–7595. 16140923 10.1158/0008-5472.CAN-05-1855

[46] Imielinski M, Berger AH, Hammerman PS, Hernandez B, Pugh TJ, et al (2012) Mapping the hallmarks of lung adenocarcinoma with massively parallel sequencing. Cell 150: 1107–1120. 22980975 10.1016/j.cell.2012.08.029 PMC3557932

[47] Andujar P, Wang J, Descatha A, Galateau-Salle F, Abd-Alsamad I, et al (2010) p16INK4A inactivation mechanisms in non-small-cell lung cancer patients occupationally exposed to asbestos. Lung Cancer 67: 23–30. 19375815 10.1016/j.lungcan.2009.03.018

[48] Blons H, Pallier K, Le Corre D, Danel C, Tremblay-Gravel M, et al (2008) Genome wide SNP comparative analysis between EGFR and KRAS mutated NSCLC and characterization of two models of oncogenic cooperation in non-small cell lung carcinoma. BMC Med Genomics 1: 25. 18549475 10.1186/1755-8794-1-25 PMC2527324

[49] The Cancer Genome Atlas (2012) Comprehensive molecular characterization of human colon and rectal cancer. Nature 487: 330–337. 22810696 10.1038/nature11252 PMC3401966

[50] Takahira T, Oda Y, Tamiya S, Yamamoto H, Kawaguchi K, et al (2004) Alterations of the p16INK4a/p14ARF pathway in clear cell sarcoma. Cancer Sci 95: 651–655. 15298727 10.1111/j.1349-7006.2004.tb03324.x PMC11158930

[51] Nagy E, Beck Z, Kiss A, Csoma E, Telek B, et al (2003) Frequent methylation of p16INK4A and p14ARF genes implicated in the evolution of chronic myeloid leukaemia from its chronic to accelerated phase. Eur J Cancer 39: 2298–2305. 14556920 10.1016/s0959-8049(03)00552-5

[52] Ueki T, Hsing AW, Gao YT, Wang BS, Shen MC, et al (2004) Alterations of p16 and prognosis in biliary tract cancers from a population-based study in China. Clin Cancer Res 10: 1717–1725. 15014024 10.1158/1078-0432.ccr-1137-3

[53] Ku CS, Wu M, Cooper DN, Naidoo N, Pawitan Y, et al (2012) Exome versus transcriptome sequencing in identifying coding region variants. Expert Rev Mol Diagn 12: 241–251. 22468815 10.1586/erm.12.10

[54] Cirulli ET, Singh A, Shianna KV, Ge D, Smith JP, et al (2010) Screening the human exome: a comparison of whole genome and whole transcriptome sequencing. Genome Biol 11: R57. 20598109 10.1186/gb-2010-11-5-r57 PMC2898068

[55] Zhu J, Sanborn JZ, Benz S, Szeto C, Hsu F, et al (2009) The UCSC Cancer Genomics Browser. Nat Methods 6: 239–240. 19333237 10.1038/nmeth0409-239 PMC5027375

