# Supplementary material for: RADIA: RNA and DNA Integrated Analysis for Somatic Mutation Detection
Source: PLoS One. 2014 Nov 18;9(11):e111516. doi: 10.1371/journal.pone.0111516 (PMC4236012; doi:10.1371/journal.pone.0111516)
Supplement: Figure S1 — Schematic of mutations detected by the DNA Only Method (DOM) and Triple BAM Method (TBM). In the first and middle columns, there is enough DNA read support for the DOM and other algorithms acting on DNA pairs to detect a mutation. In the middle and last columns, there is sufficient RNA read support for the TBM to detect a mutation. The middle column illustrates “RNA Confirmation” mutations that are detected by both the DOM and the TBM due to high read support in both the DNA and RNA. The last column represents the “RNA Rescue” mutations that have some support in the DNA and strong evidence in the RNA. The RNA Rescue mutations are typically missed by traditional mutation calling algorithms that only investigate the pairs of DNA. (PDF) [file pone.0111516.s001.pdf]

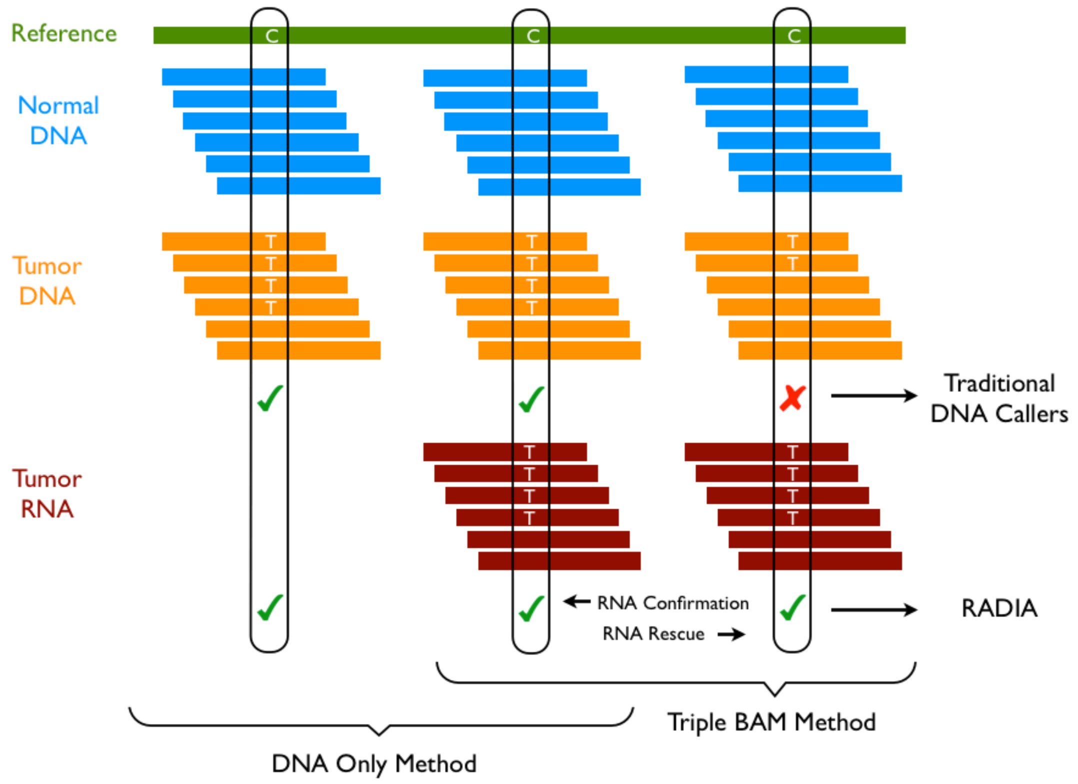

Figure S1: Schematic of mutations detected by the DNA Only Method (DOM) and Triple BAM Method (TBM). In the first and middle columns, there is enough DNA read support for the DOM and other algorithms acting on DNA pairs to detect a mutation. In the middle and last columns, there is sufficient RNA read support for the TBM to detect a mutation. The middle column illustrates “RNA Confirmation” mutations that are detected by both the DOM and the TBM due to high read support in both the DNA and RNA. The last column represents the “RNA Rescue” mutations that have some support in the DNA and strong evidence in the RNA. The RNA Rescue mutations are typically missed by traditional mutation calling algorithms that only investigate the pairs of DNA.
