# Supplementary material for: RADIA: RNA and DNA Integrated Analysis for Somatic Mutation Detection
Source: PLoS One. 2014 Nov 18;9(11):e111516. doi: 10.1371/journal.pone.0111516 (PMC4236012; doi:10.1371/journal.pone.0111516)
Supplement: Figure S2 — Diagram of bamsurgeon methodology. Mutations are spiked into BAM files by selecting locations with adequate coverage, extracting the reads, and adjusting the VAF according to the desirable VAF distribution. Once the bases in the reads are changed, the reads are remapped to the genome, replacing the reads in the original BAM file. (PDF) [file pone.0111516.s002.pdf]

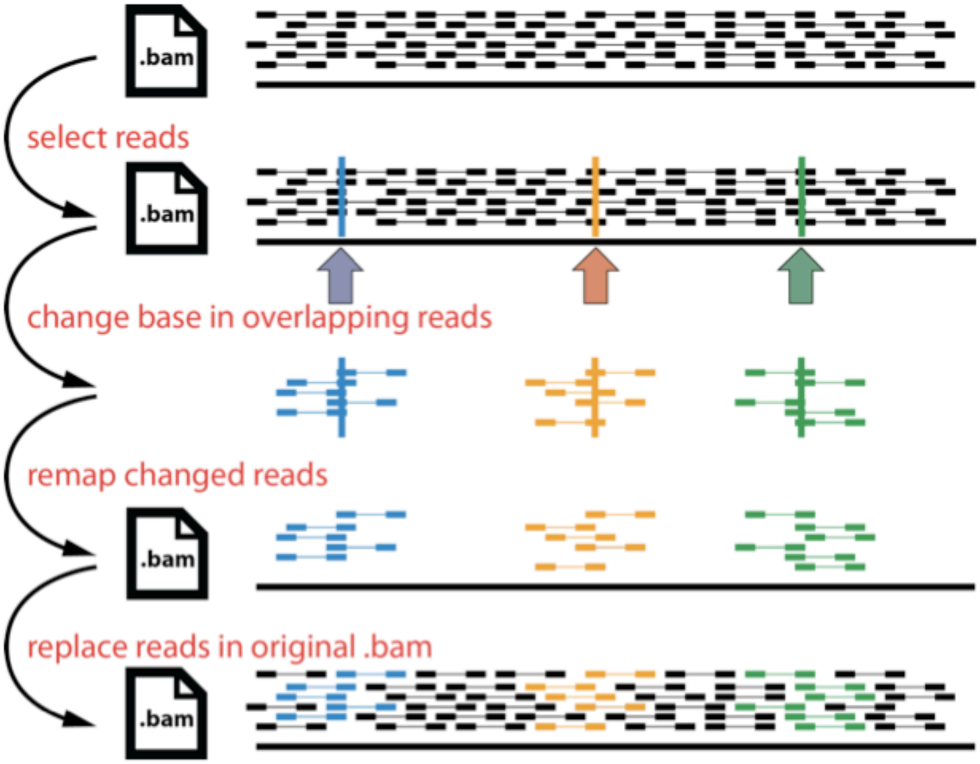

Figure S2: Diagram of bamsurgeon methodology. Mutations are spiked into BAM files by selecting locations with adequate coverage, extracting the reads, and adjusting the VAF according to the desirable VAF distribution. Once the bases in the reads are changed, the reads are remapped to the genome, replacing the reads in the original BAM file.
