# Supplementary material for: RADIA: RNA and DNA Integrated Analysis for Somatic Mutation Detection
Source: PLoS One. 2014 Nov 18;9(11):e111516. doi: 10.1371/journal.pone.0111516 (PMC4236012; doi:10.1371/journal.pone.0111516)
Supplement: Figure S3 — Filters applied in the Variable DNA-Constant RNA bamsurgeon simulation experiment. The DNA variant allele frequencies were distributed from 1–50% and the RNA was held constant at 25%. Most of the DOM mutations were filtered because of the low variant allele frequency and tumor strand bias. In the TBM, most of the mutations were filtered due to the minimum number of alternative alleles required to make a call (n = 4) and strand bias in the tumor DNA and RNA. (PDF) [file pone.0111516.s003.pdf]

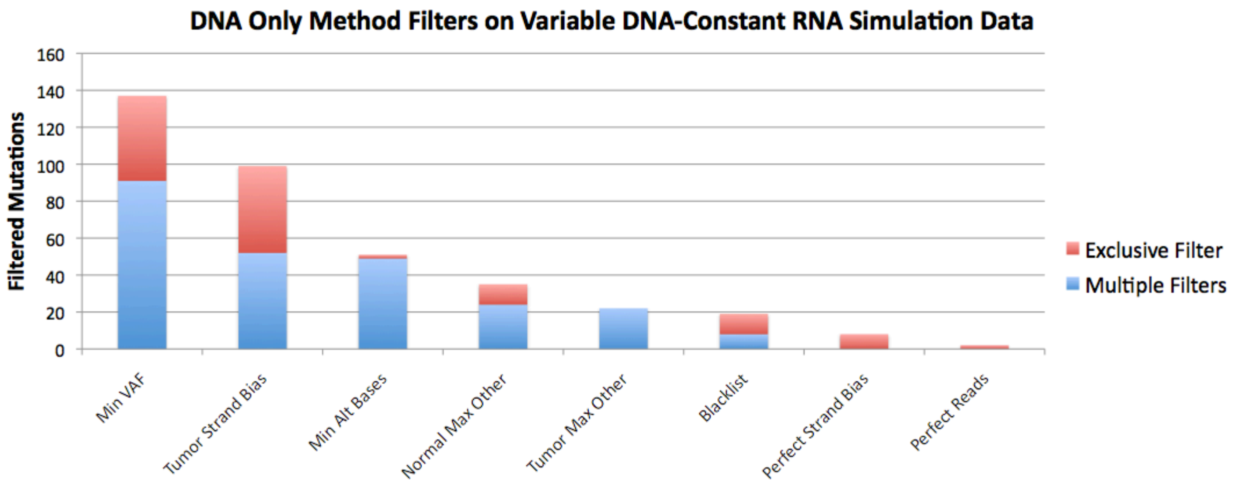

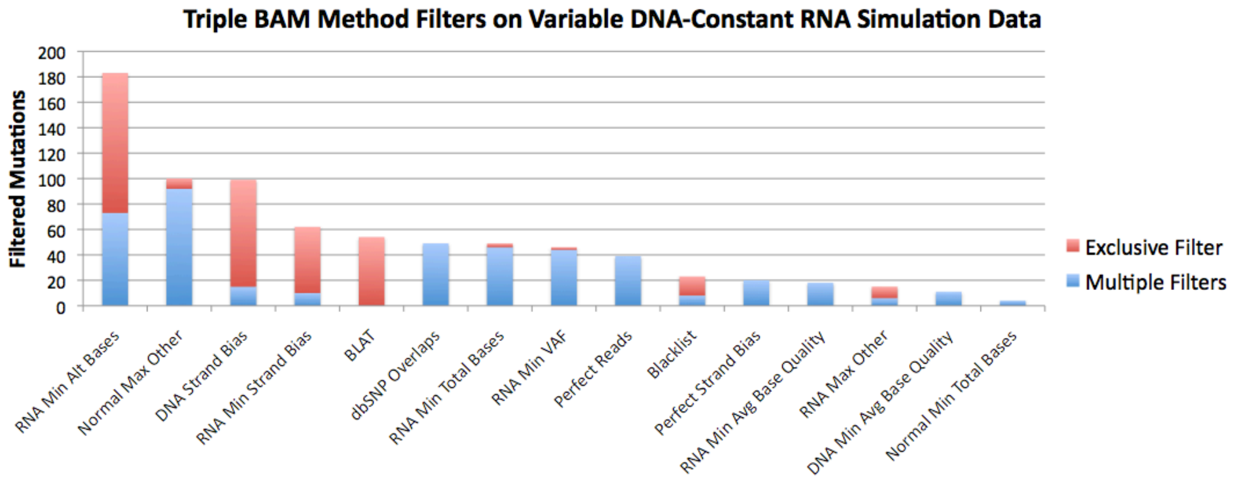

Figure S3: Filters applied in the Variable DNA-Constant RNA bamsurgeon simulation experiment. The DNA variant allele frequencies were distributed from 1-50% and the RNA was held constant at 25%. Most of the DOM mutations were filtered because of the low variant allele frequency and tumor strand bias. In the TBM, most of the mutations were filtered due to the minimum number of alternative alleles required to make a call (n=4) and strand bias in the tumor DNA and RNA.
