# Supplementary material for: RADIA: RNA and DNA Integrated Analysis for Somatic Mutation Detection
Source: PLoS One. 2014 Nov 18;9(11):e111516. doi: 10.1371/journal.pone.0111516 (PMC4236012; doi:10.1371/journal.pone.0111516)
Supplement: Figure S4 — Filters applied in the Low Frequency DNA-Variable RNA bamsurgeon simulation experiment. The RNA variant allele frequencies were distributed from 1–50% and the DNA was held at 10% or less. Most of the DOM mutations were filtered because of the low DNA variant allele frequency and tumor strand bias. In the TBM, most of the mutations were filtered due to the minimum number of alternative alleles required to make a call (n = 4) and the low RNA variant allele frequency. (PDF) [file pone.0111516.s004.pdf]

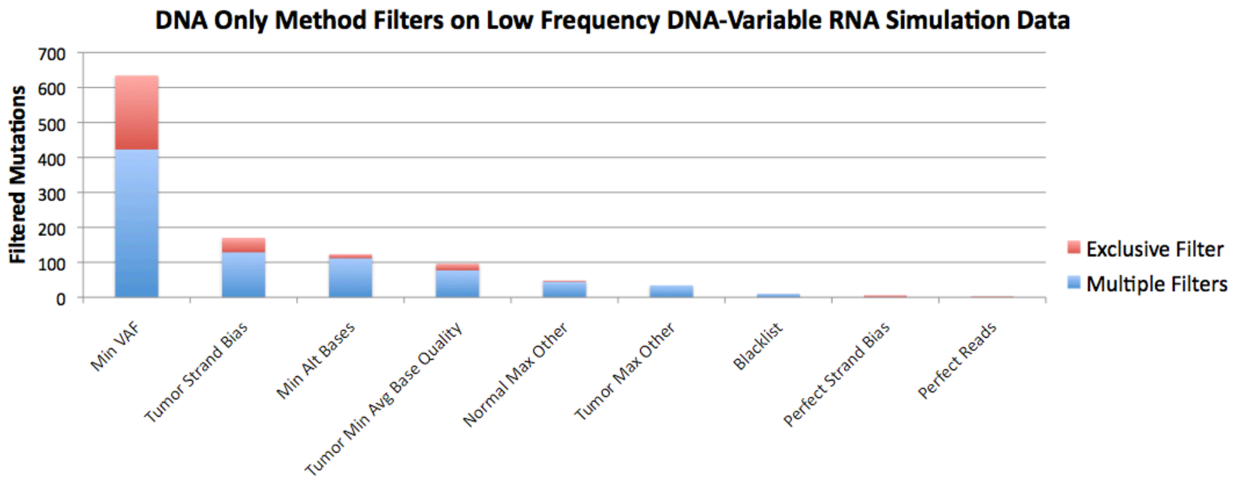

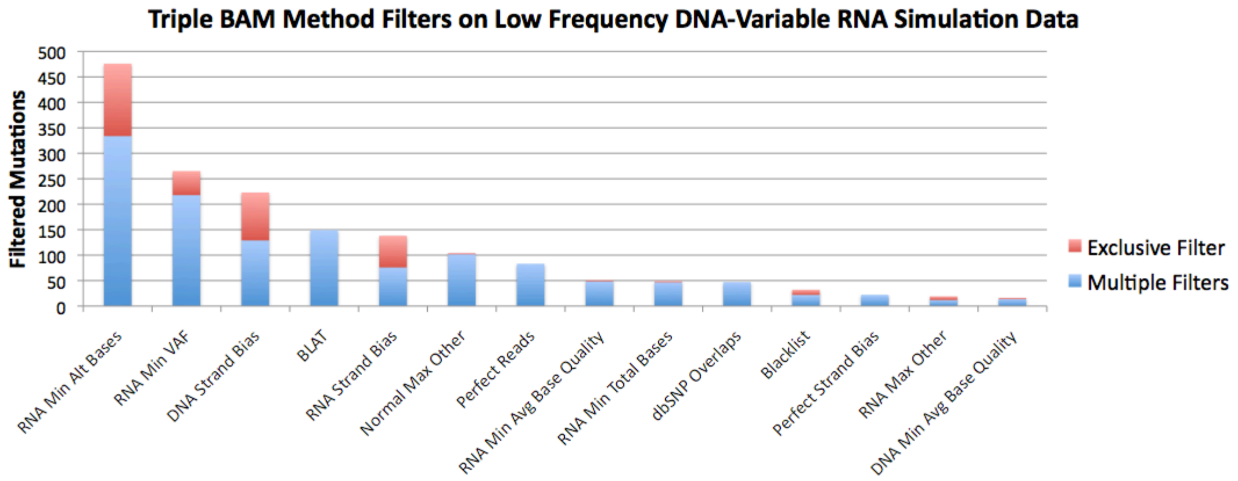

Figure S4: Filters applied in the Low Frequency DNA-Variable RNA bamsurgeon simulation experiment. The RNA variant allele frequencies were distributed from 1-50% and the DNA was held at 10% or less. Most of the DOM mutations were filtered because of the low DNA variant allele frequency and tumor strand bias. In the TBM, most of the mutations were filtered due to the minimum number of alternative alleles required to make a call (n=4) and the low RNA variant allele frequency.
