# Supplementary material for: RADIA: RNA and DNA Integrated Analysis for Somatic Mutation Detection
Source: PLoS One. 2014 Nov 18;9(11):e111516. doi: 10.1371/journal.pone.0111516 (PMC4236012; doi:10.1371/journal.pone.0111516)
Supplement: Figure S5 — Distribution of overlaps between RADIA and the endometrial TCGA MAF file. The distribution of the overlaps between RADIA and the validated somatic mutations from the endometrial TCGA network MAF file. (PDF) [file pone.0111516.s005.pdf]

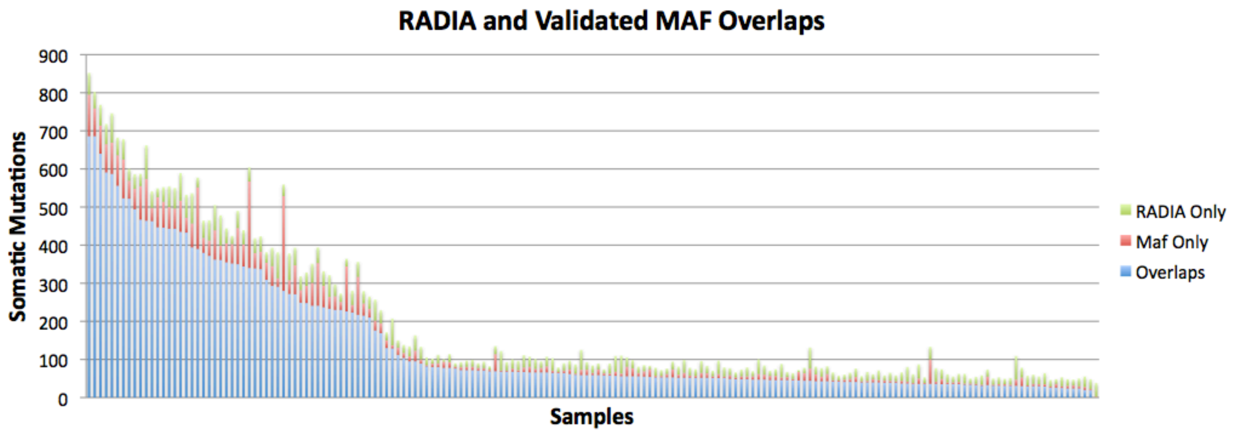

Figure S5: Distribution of overlaps between RADIA and the endometrial TCGA MAF file. The distribution of the overlaps between RADIA and the validated somatic mutations from the endometrial TCGA network MAF file.
