# Supplementary material for: RADIA: RNA and DNA Integrated Analysis for Somatic Mutation Detection
Source: PLoS One. 2014 Nov 18;9(11):e111516. doi: 10.1371/journal.pone.0111516 (PMC4236012; doi:10.1371/journal.pone.0111516)
Supplement: Figure S6 — Filters applied to the RADIA mutations that validated as somatic in the endometrial TCGA MAF file. Thirty-three percent of the mutations had a DNA VAF of eight percent or less while 23% landed in blacklist regions that were ignored. (PDF) [file pone.0111516.s006.pdf]

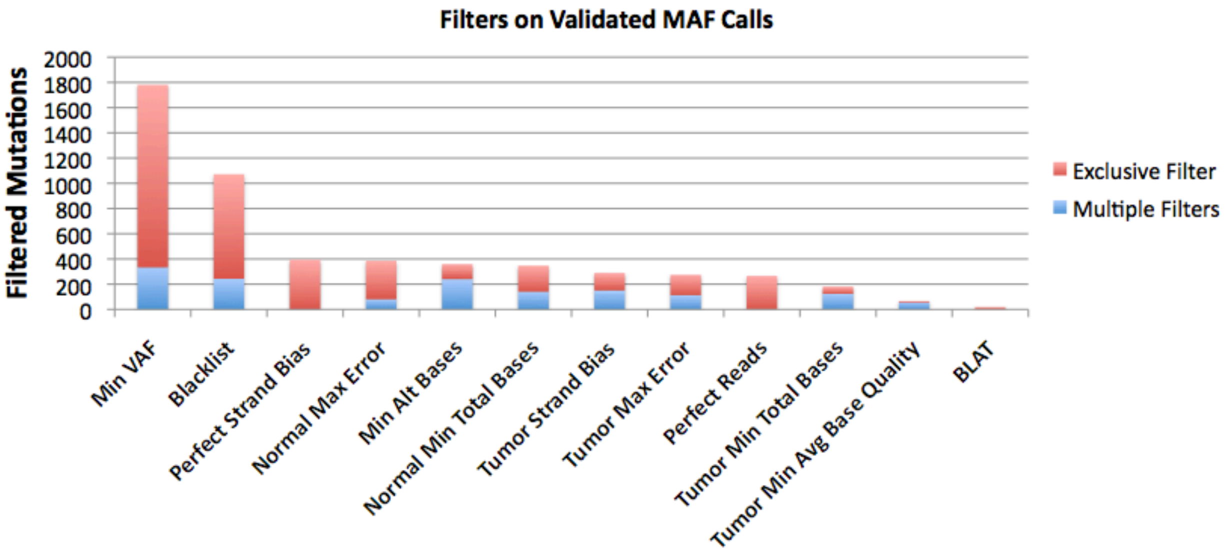

Figure S6: Filters applied to the RADIA mutations that validated as somatic in the endometrial TCGA MAF file. Thirty-three percent of the mutations had a DNA VAF of eight percent or less while 23% landed in blacklist regions that were ignored.
