# Supplementary material for: RADIA: RNA and DNA Integrated Analysis for Somatic Mutation Detection
Source: PLoS One. 2014 Nov 18;9(11):e111516. doi: 10.1371/journal.pone.0111516 (PMC4236012; doi:10.1371/journal.pone.0111516)
Supplement: Figure S7 — RNA Rescue mutations are primarily at low DNA VAFs. RNA Rescue mutations are primarily found at low DNA variant allele frequencies, but they also occur at higher frequencies where they were filtered due to non-depth related artifacts (e.g. strand-bias). (PDF) [file pone.0111516.s007.pdf]

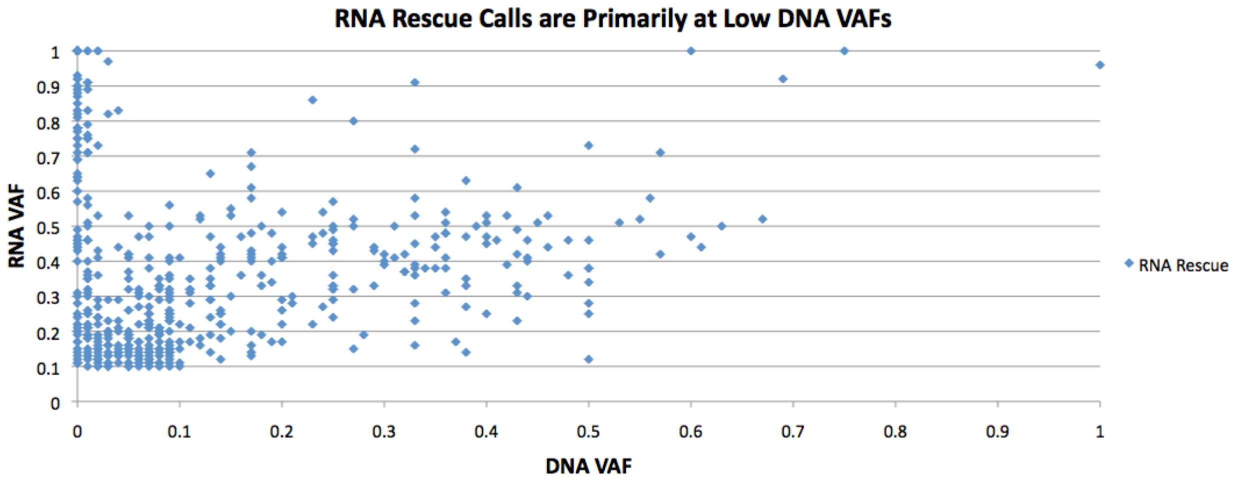

Figure S7: RNA Rescue calls are primarily at low DNA VAFs. RNA Rescue calls are primarily found at low DNA variant allele frequencies, but they also occur at higher frequencies where they were filtered due to non-depth related artifacts (e.g. strand-bias).
