# Supplementary material for: RADIA: RNA and DNA Integrated Analysis for Somatic Mutation Detection
Source: PLoS One. 2014 Nov 18;9(11):e111516. doi: 10.1371/journal.pone.0111516 (PMC4236012; doi:10.1371/journal.pone.0111516)
Supplement: Figure S8 — Distribution of RNA Confirmation Calls. The total number of mutations (blue) that are covered by at least one RNA read (yellow), one RNA read supporting the alternative allele (orange), and RNA Confirmation mutations with high support in both the DNA and RNA (purple). (PDF) [file pone.0111516.s008.pdf]

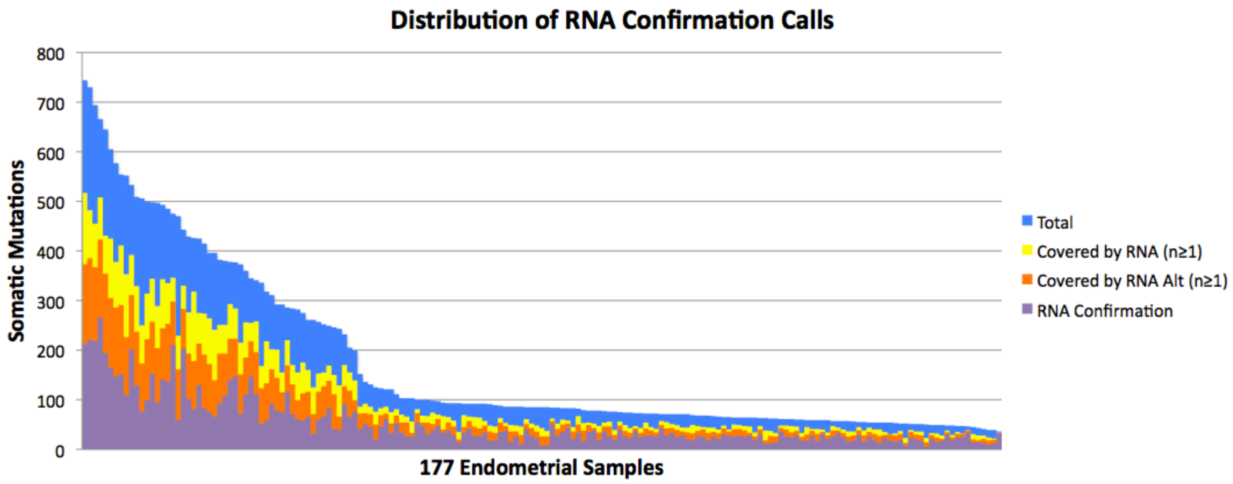

Figure S8: Distribution of RNA Confirmation Calls. The total number of mutations (blue) that are covered by at least one RNA read (yellow), one RNA read supporting the alternative allele (orange), and RNA Confirmation mutations with high support in both the DNA and RNA (purple).
