# Supplementary material for: RADIA: RNA and DNA Integrated Analysis for Somatic Mutation Detection
Source: PLoS One. 2014 Nov 18;9(11):e111516. doi: 10.1371/journal.pone.0111516 (PMC4236012; doi:10.1371/journal.pone.0111516)
Supplement: Table S1 — Summary of TCGA samples analyzed by RADIA. RADIA has been run on over 3,300 TCGA samples across 15 different types of cancer. The RNA Rescue mutations make up two to seven percent of the total somatic mutations across the 15 types of cancer. Variant Call Format (VCF) and Mutation Annotation Format (MAF) files can be downloaded from the TCGA Data Portal (https://tcga-data.nci.nih.gov/tcga/). Open-access somatic MAFs can be visualized and downloaded via the UCSC Cancer Browser (https://genome-cancer.ucsc.edu/). (PDF) [file pone.0111516.s009.pdf]

Cancer Type	Sample Count	Total Somatic SNVs	DNA Only Method	Triple BAM Method	RNA Rescue Calls	RNA Rescue Percent
Uterine Corpus Endometrioid Carcinoma	177	27900	27390	6325	510	2%
Lung Adenocarcinoma	263	85044	79347	21484	5697	7%
Kidney Chromophobe	66	4163	3957	1042	206	5%
Thyroid Carcinoma	430	20849	19836	2882	1013	5%
Skin Cutaneous Melanoma	347	584431	573925	70091	10498	2%
Low-Grade Glioma	289	13852	12837	3926	1015	4%
Prostate Adenocarcinoma	314	14630	12653	4631	846	6%
Kidney Renal Papillary Cell Carcinoma	196	28662	27346	6334	1316	5%
Adrenocortical Carcinoma	91	9891	9748	1863	143	2%

Cancer Type	Sample Count	Total Somatic SNVs	DNA Only Method	Triple BAM Method	RNA Rescue Calls	RNA Rescue Percent
Uterine Carcinosarcoma	57	8987	8776	2832	211	2%
Stomach Adenocarcinoma	327	134,895	127,405	51,596	7,490	6%
Liver Hepatocellular Carcinoma	236	32,854	32,113	7,056	741	2%
Cervical Squamous Cell Carcinoma and Endocervical Adenocarcinoma	202	62,252	59,835	21,485	2,417	4%
Pheochromocytoma and Paraganglioma	187	2,993	2,820	761	173	6%
Pancreatic Adenocarcinoma	137	23,692	22,850	9,653	842	4%

Table S1: Summary of TCGA samples analyzed by RADIA. RADIA has been run on over 3,300 TCGA samples across 15 different types of cancer. The RNA Rescue mutations make up two to seven percent of the total somatic mutations across the 15 types of cancer. Variant Call Format (VCF) and Mutation Annotation Format (MAF) files can be downloaded from the TCGA Data Portal (https://tcga-data.nci.nih.gov/tcga/). Open-access somatic MAFs can be visualized and downloaded via the UCSC Cancer Browser (https://genome-cancer.ucsc.edu/).
